# Annual Commencement of the Baltimore College of Dental Surgery

**Published:** 1852-04

**Authors:** 


### EDITORIAL DEPARTMENT.

***Annual Commencement of the Baltimore College of Dental Surgery.***—The exercises of the last Commencement were held in the hall of the college on the evening of March 18th, 1852, in the presence of a large audience of ladies and gentlemen, and the examining committee. This committee, it may be necessary to state, is composed of three medical gentlemen and four dentists, who have been appointed from year to year, since the first recommendation of the American Medical Association. They take part in the examinations, and no degree is conferred without their approbation.

The exercises were opened by prayer, by the Rev. B. H. Nadal. The following candidates were then called up by the dean, Professor W. R. Handy, and the degrees conferred by Prof. C. A. Harris.

*Graduates of Session* 1851--'2.

| | Residence. | Thesis. |
|---|---|---|
| Thos. D. Siminton, | Pa., | Civilized and Uncivilized Man. |
| Aldabert J. Volck, | Germany, | Dental Apparatus of the Nerves. |
| George Mears, | Pa., | Preservation of the Teeth. |
| P. Henry McCargo, | Va., | Caries of the Teeth. |
| Stanhope A. Sudderth, | N. C., | Caries of the Teeth. |
| Warren Welch, | Md., | Dental Caries. |
| Horace E. Chapin, | Mass., | Mechanical Dentistry. |
| Francis E. Clautier, | La., | Caries of the Teeth. |
| Richard H. Finch, | Va. | Anasthesia. |
| Henry F. Stevens, | Conn. | Deciduous Teeth. |
| John A. Cobbs, | Va. | Diseases of the Maxillary Sinus. |
| Albert A. Cleaveland, | Mass. | Dyspepsia. |

Dr. Eleazar Parmly of New York, the provost of the college, now arose and addressed the graduates and audience on the subject of dental education, a report of which will be found in another part of the present number of the Journal. We ask for it the perusal of our readers. The Doctor takes a plain, common sense practical view of the subject.

Professor Robert Arthur, of Washington city, the orator of the occasion, pronounced the *valedictory*. This contains most excellent advise to the graduating class, warning them of the difficulties in their path, and encouraging them to perseverance, and with unflinching firmness to remain true to themselves, true to their professional brethren, and true to their patients, as the safest, surest, and most lasting way to secure the approbation of their own consciences, pecuniary reward, and the approbation of their fellow men. But we find it impossible to give even an outline of this excellent address, and we would be glad to see it published, not only for the gratification of the graduates, for whom it was especially designed, but also for the profession generally.

The exercises were interspersed with appropriate music at proper intervals. At the conclusion, the audience was dismissed with a benediction from the Rev. B. H. Nadal.

